# Machiavellian Ways to Academic Cheating: A Mediational and Interactional Model

**DOI:** 10.3389/fpsyg.2018.00695

**Published:** 2018-05-14

**Authors:** Claudio Barbaranelli, Maria L. Farnese, Carlo Tramontano, Roberta Fida, Valerio Ghezzi, Marinella Paciello, Philip Long

**Affiliations:** ^1^Department of Psychology, Sapienza University of Rome, Rome, Italy; ^2^Centre for Advances in Behavioural Science, Coventry University, Coventry, United Kingdom; ^3^Norwich Business School, University of East Anglia, Norwich, United Kingdom; ^4^Faculty of Psychology, Università Telematica Internazionale Uninettuno, Rome, Italy; ^5^Centre for Staff and Educational Development, University of East Anglia, Norwich, United Kingdom

**Keywords:** Machiavellianism, academic cheating, moral disengagement, approach to learning, normative cheating

## Abstract

Academic cheating has become a pervasive practice from primary schools to university. This study aims at investigating this phenomenon through a nomological network which integrates different theoretical frameworks and models, such as trait and social-cognitive theories and models regarding the approaches to learning and contextual/normative environment. Results on a sample of more than 200 Italian university students show that the Amoral Manipulation facet of Machiavellianism, Academic Moral Disengagement, Deep Approach to Learning, and Normative Academic Cheating are significantly associated with Individual Academic Cheating. Moreover, results show a significant latent interaction effect between Normative Academic Cheating and Amoral Manipulation Machiavellianism: “amoral Machiavellians” students are more prone to resort to Academic Cheating in contexts where Academic Cheating is adopted as a practice by their peers, while this effect is not significant in contexts where Academic Cheating is not normative. Results also show that Academic Moral Disengagement and Deep Approach to learning partially mediate the relationship between Amoral Manipulation and Academic Cheating. Practical implications of these results are discussed.

## Introduction

Academic cheating has become a pervasive practice, from primary schools to university (McCabe and Treviño, 1997; Murdock et al., 2001; McCabe et al., 2012). The negative consequences of this phenomenon are easy to envisage. Indeed, engagement in academic dishonesty may increase the likelihood of misconduct at work (e.g., Wowra, 2007a; McCabe et al., 2012). It may also result in the “normalization” of unethical behavior across contexts by “fostering a mindset that predisposes individuals to engage in this kind of behavior” (Fida et al., 2016, p. 2). Moreover, the pervasiveness of academic cheating may likely result in damage for the labor market in which graduates will enter, since academic degrees gained by cheating may jeopardize their validity.

**FIGURE 1:**
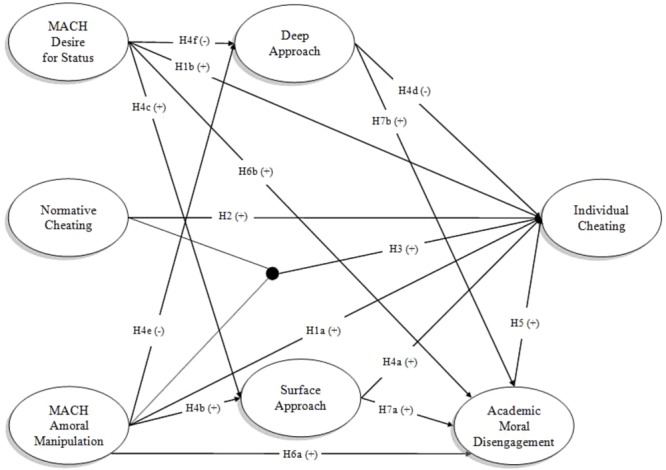
The hypothesized model. For sake of clarity, covariances among Desire for Status and Amoral Manipulation, and among Surface Approach and Deep Approach were not drawn.

This study is aimed at examining a nomological network for the understanding of cheating behaviors in the academic context. Specifically, by integrating different theoretical frameworks, we considered multiple mediators and a moderator of academic cheating behaviors (**Figure 1**). First, since academic cheating is a form of deviant behavior (Murdock and Anderman, 2006), we explored the role of Machiavellianism, an important determinant of such type of conduct (DeShong et al., 2017). Furthermore, we included two relevant mediators: approach to learning (Fleming, 1996; Xin, 2011) and moral disengagement (Bandura, 2016). The former has been included because of the specific context under study. Indeed, since we are investigating misbehavior that are in complete contraposition with “learning,” the core element of the academic institutions, we decided to explore the role of two distinct motivational orientations toward studying, that are surface or deep learning. Moral disengagement has been included, in line with Bandura’s (1991, 2016) theoretical framework on moral agency, as an important proximal antecedent of deviant behavior. Indeed, the disinhibitory power of moral disengagement has been highlighted in relation to a wide variety of misbehavior in a range of contexts, including cheating behavior in higher education (Fida et al., 2016).

In addition, by adopting an interactionist perspective (Reynolds et al., 2010), we also considered a potential moderator to better understand the association of Machiavellianism with the engagement in cheating behavior. Specifically, we examined perception of peers’ involvement in cheating behaviors, as a measure of the implicit social norm and of the “permeability of the context to unethical conducts” (Farnese et al., 2011, p. 357). In the following sections we address the issue of academic cheating behavior and detail the rationale for including these specific variables and for hypothesizing the suggested paths.

### Academic Cheating Behaviors

Academic cheating behavior refers to a wide range of deviant conducts in which students may engage during their vocational education (Murdock and Anderman, 2006; McCabe et al., 2012). The reported incidence of cheating in the literature (e.g., Whitley, 1998; McCabe et al., 2012; International Center for Academic Integrity [ICAI], 2015) speaks of a phenomenon that is other than marginal and seems to be increasingly pervasive, especially when considering that the available data are expected to provide only an underestimate picture of a behavior that is by its nature covert and underreported. Furthermore, academic cheating is neither confined in some cultures nor prevalent in specific higher education programs. Indeed, there are studies reporting and investigating academic dishonesty in a wide range of countries worldwide – including but not limited to China and Thailand (Yang et al., 2017), New Zealand (Henning et al., 2013), Sweden (Trost, 2009) – as well as across faculties (see Krueger, 2014).

Beside the investigation of prevalence and incidence of cheating behavior, academic research has been largely oriented toward the identification and the understanding of potential factors fostering or hindering it. In particular, an overview of the most relevant literature suggests the pivotal role of some individual differences. First: personality traits. A recent meta-analytic review, examining the relationship between the Big Five and academic dishonesty, provided evidence for the importance of conscientiousness and agreeableness (Giluk and Postlethwaite, 2015). Furthermore, the so-called Dark Triad of personality – a label referring to psychopathy, narcissism, and Machiavellianism traits – has been extensively studied in relation to cheating behaviors (e.g., Jonason et al., 2014; Roeser et al., 2016; Dane et al., 2018). With specific reference to dishonesty in the academic setting, evidences have been provided for the impact of narcissism (e.g., Brunell et al., 2011; Menon and Sharland, 2011), and even more strongly for the relevance of Machiavellianism (e.g., Wowra, 2007a; Bloodgood et al., 2010).

Second: motivation. In particular, drawing on the goal orientation and self-determination theories (e.g., Deci and Ryan, 1985; Ames and Archer, 1988), several researches showed that performance-oriented and extrinsic-motivated students are more likely to cheat than mastery-oriented and intrinsic-motivated ones (e.g., Anderman et al., 1998; Jordan, 2001; Murdock et al., 2001; Baker, 2004; Van Yperen et al., 2011).

Third: morality. Although the adoption of an Honor Code has been suggested as a key institutional factor to prevent academic cheating behavior (e.g., McCabe et al., 2012), an extensive literature has underlined that the mere existence of an explicit and formalized set of ethical conducts is not *per se* sufficient (e.g., Scanlan, 2006; Bing et al., 2012; O’Neill and Pfeiffer, 2012; Popoola et al., 2017). Indeed, academic research provides evidence about the need of considering key dimensions related to the individual moral domain, such as cognitive dissonance and attitude toward cheating (e.g., Storch and Storch, 2003), moral character (Wray et al., 2016), moral identity (Wowra, 2007b), and neutralization techniques (Curasi, 2013; Olafson et al., 2013). In particular, a recently published study (Fida et al., 2016) highlighted the longitudinal interconnection between the engagement in cheating behavior and students’ moral disengagement. Specifically, over time cognitive mechanisms aimed at temporarily deactivating students’ moral control (i.e., moral disengagement) allow the engagement in cheating behavior, which in turn facilitates the activation of those same mechanisms.

Finally: perception of peers’ behavior. Students are immersed in a social environment rather than being isolated “monads”; hence, the understanding of their behavior cannot disregard their perception of the context. Research has shown that witnessing others’ cheating may facilitate the engagement in similar misconduct, as a result of the assumption that such behavior should not be considered particularly reprehensible or even advantageous (e.g., O’Rourke et al., 2010; Bernardi et al., 2012). Indeed, the mere students’ perception that others adopt dishonest strategies has resulted to be associated with cheating behavior (e.g., McCabe et al., 2001, 2006; Yang et al., 2017), and has been claimed to be one of the most relevant factors (e.g., McCabe et al., 2012).

### Role of Machiavellianism

Machiavellianism is a personality construct introduced in the literature by Christie and Geis (1970). It refers to a personality pattern characterizing people motivated by the maximization of their own interests, goals, and needs, with spare regard toward the negative effects this may cause to others. According to Christie and Geis (1970), Machiavellianism is a multidimensional construct whose key features are: (a) manipulating others, (b) lack or disregard of conventional morality, and (c) cynical view of the world and of human nature.

Several studies explored the relationship of Machiavellianism with different constructs in a variety of populations. Overall, this literature highlighted the correlation of Machiavellianism with different deviant and antisocial conducts, such as occupational problems, negative and counterproductive work behaviors, and cheating [for a review see DeShong et al. (2017) and Jones and Paulhus (2009)].

Mach IV scale, originally developed by Christie and Geis (1970), still remains one of the most used instruments to assess Machiavellianism. However, this scale has been subject to criticisms as far as its psychometric characteristics are concerned (see Dahling et al., 2009). While the construct has been conceived as multidimensional, and the scale, consistently, measures the three aforementioned distinct aspects (i.e., interpersonal tactics, cynical view of human nature, and disregard for conventional morality), it has been substantially used as a unidimensional measure.

Dahling et al. (2009) proposed an alternative multidimensional conceptualization of Machiavellianism, focusing on four different facets: (a) amoral manipulation, (b) distrust of others, (c) desire for control, and (d) desire for status. They also developed a new measure, the Machiavellianism Personality Scale (MPS), composed by four subscales corresponding to the different facets of Machiavellianism defined above. According to the authors, these four dimensions represent different although interrelated manifestations of a same overarching construct, showing similar relations with its antecedents and consequences. In particular, a central aspect of Machiavellians is built upon the original conceptualization of the construct, which put at the heart of it an approach characterized by a deliberate tendency toward manipulating and betraying others whenever the opportunity of gaining from this behavior is presented. Accordingly, the *Amoral Manipulation* facet is defined “as a willingness to disregard standards of morality and see value in behaviors that benefit the self at the expense of others” (Dahling et al., 2009, p. 228). Furthermore, Machiavellians have a negative outlook toward others: in particular, they actively attribute to others hostile intentions and motivations. Accordingly, another facet of Machiavellianism is *Distrust of Others* defined as “a cynical outlook on the motivations and intentions of others with a concern for the negative implications that those intentions have for the self” (Dahling et al., 2009, p. 227). A third aspect addressed by the MPS is concerned with the desire of dominating interpersonal situations due to the fact that Machiavellians see others as a threat to the achievement of their goals and needs. Accordingly, the *Desire for Control* facet is defined as “a need to exercise dominance over interpersonal situations to minimize the extent to which others have power” (Dahling et al., 2009, p. 228). Finally, the fourth facet considered by Dahling et al. (2009) has to do with the aims and goals that Machiavellians strive to pursuit: indeed, they put value on external and extrinsic goals, such as status, power, and wealth, while they disregard intrinsic and internal goals such as individual and personal fulfillment. Thus, the *Desire for Status* facet is defined “as a desire to accumulate external indicators of success” (Dahling et al., 2009, p. 228).

It is not surprising that the relationship of Machiavellianism with cheating in academic settings has been object of study, given the manipulative and amoral tendencies of Machiavellians, but findings are indeed quite inconclusive. While some studies evidenced a positive relationship between Machiavellianism and cheating (e.g., Bogart et al., 1970; Williams et al., 2010) others did not replicate this result (e.g., Flynn et al., 1987; Cizek, 1999). However, failures in showing this association have been attributed to weak methodological research designs or to weak Machiavellianism measures used (Dahling et al., 2009).

When considering the multifaceted nature of Machiavellianism, it may be questioned the reason why all facets of this complex construct should be expected to be actually connected with academic cheating. Indeed, the *distrust of others* and *desire for control* facets of Machiavellianism are mainly focused on the interpersonal features of the construct, and as such there is not a clear rationale for positing any association with academic cheating. On the contrary, since cheating behavior has to do with violation of written (or unwritten) moral laws, the amoral manipulation and desire for status seem to be key dimensions. In particular, the amoral-manipulative facet of Machiavellianism could be positively related with the propensity to cheat in academic contexts. More specifically, when the opportunity to cheat and lie is given (for instance in case of scarce anticipated consequences derived from cheating or of poor surveillance during tests), Machiavellians will engage in academic misconducts, due to their proneness to disregard norms and rules for their own benefit. Indeed, as noted by Cooper and Peterson (1980) Machiavellians “would not need any personal inducement to do so, just the likelihood of not getting caught” (p. 71). Similarly, since the attainment of good grades can be related to achieving external recognition in terms of extrinsic goals, one may hypothesize that students high in desire for status could more likely adopt cheating behavior. Accordingly, we focused our study on the latter two facets and we formulate the following hypothesis:

*H1: Amoral manipulation (H1a) and Desire for Status (H1b) are positively associated to individual academic cheating*.

### Context as a Source of Norms for Cheating

Literature has attested that the social environment exerts an influence on students’ attitudes and behavior toward cheating (Broeckelman-Post, 2008; Carrell et al., 2008; Farnese et al., 2011). Some scholars defined *cheating culture* as a context where cheating behaviors are tolerated, and where people believe that cheating is needed for achieving a goal and share the perception that everyone is cheating to this end (Crittenden et al., 2009). This culture represents a source of norms that – explicitly, but also and above all implicitly: (a) defines the organizational (or group) system of values and priorities; (b) supports the adherence to shared moral codes; and (c) defines routines, habits, and practices molding consequently individuals’ beliefs, attitudes, choices, and actions (Schein, 1985). Research showed that a cheating culture may influence propensity to dishonesty (Crittenden et al., 2009) and discourage peer reporting (McCabe et al., 2001). Overall, it weakens respect for integrity as a value (Treviño, 1990; Paine, 1994) and enhances the perceived permeability of the context to unethical conducts (Farnese et al., 2011).

Specifically, peers’ attitudes and behavior are identified as prominent precursors of academic cheating as they provide a model for the expectancies on students’ role and responsibilities (see role theory, Katz and Kahn, 1978). Further, in line with the reinforcement theory (Luthans and Kreitner, 1985), observing peers’ behavior shows the possible positive (e.g., advantages, rewards) or negative (e.g., sanctions) consequences associated with engaging cheating behavior. Accordingly, we formulate the following hypothesis:

H2: Perception of academic cheating as normative among peers is positively associated to individual academic cheating.

This twofold nature of peers cheating (i.e., both a norm and a model) may further represent a “culture milieu” where the relationship between Machiavellianism and cheating may grow and strengthen. Students high in Machiavellianism may express an affective detachment and lack of concern for conventional morality (the so-called “cool syndrome”, e.g., Flynn et al., 1987) and an opportunistic approach, which makes easier for them to adopt unethical behaviors when the context (i.e., the *group culture*) does not sanction or even legitimate these behaviors. Specifically, adopting the rational choice perspective (Piliavin et al., 1986; Cornish, 1993; McCabe et al., 2001), Machiavellians students may perceive that they are in a “low-risk” setting when: (a) many colleagues share the implicit norm that cheating is tolerated or even is the most functional way to pursue their goals; and (b) negative consequences of cheating are modest because the probability of being caught is low, or sanctions are not adopted. These contextual clues enhance their propensity to act cheating behaviors. Overall, perception of a widespread unethical culture among peers could reinforce selfish acts in Machiavellians students, leading them to not care about playing the rules and to adopt selfish behaviors in order, for example, to obtain advantages (e.g., pass an examination), or do not lose benefits compared to colleagues (who, by behaving illicitly, could obtain more advantages).

Empirical evidences showed that the perception of peer cheating not only influences individual cheating behavior, but also increases the likelihood of engaging in academic misconduct leading to multiplicative social effect (Carrell et al., 2008) or a cumulative effect, due to the splitting of spoils (Wiltermuth, 2011). Overall, Machiavellians students’ opportunistic approach may lead them to consider that, in unethical contexts, they can obtain higher direct or indirect selfish advantages by behaving consistently with their environment. That is, the more they perceive that their peers engage in cheating behavior, the more likely they may do the same. Thus, we formulate the following hypothesis:

H3: When academic cheating is perceived as normative (i.e., high perception of others cheating), students with high scores in Amoral Machiavellianism will engage more frequently in academic cheating, than when academic cheating is not normative.

### Role of Surface and Deep Approach to Learning

In their classic papers on deep and surface learning, Marton and Saljo (1976) differentiated between an approach to learning which essentially relied on reproducing knowledge and one which required students to transform knowledge: the first approach they termed surface learning and the second they termed deep learning. Since the publication of this seminal research, the concept of deep and surface approaches to learning has become a widely accepted model of how students approach the task of learning in higher education (Beattie et al., 1997; Rust, 2002). While there is an on-going discussion within the academic literature around the extent to which a student’s approach to learning is a product of their attributes (such as their personality and their motivation) or their personal characteristics (such as IQ or cognitive style), or their intention or the extent to which the students exhibited serialist or holist approaches to their learning, what appears to be generally agreed on is that the terms Deep and Surface approaches to learning have meaning and significance which manifest themselves in the field of assessment (Scouller, 1998). In reality the idea that there is a clear cut distinction between those students who adopt a deep approach to their learning and those adopting a surface approach ignores the extent to which student behavior varies dependent on a number of variables, leading Entwistle (1992) to promote a third option which he termed the strategic approach in which students, while displaying a predominantly deep or surface approach will, nevertheless, use whatever approach is seen as most likely to secure the highest mark.

That there is a clear relationship between the design of assessment tasks and the approaches to learning most likely to be adopted by the student is well documented (Trigwell and Prosser, 1991; Byrne et al., 2002; Gijbels et al., 2005). Weller et al. (2013) have argued that the design of the assessment task should be such that it requires the student to adopt a deep approach if they are to be successful. In his review of accountancy students and the factors which increase their predisposition to cheat in assessment, Fleming (1996) has identified a strong correlation between those students adopting a deep approach to study being less likely to cheat, and those adopting a surface approach being more likely to cheat. Xin (2011) goes so far as to suggest that “… the extant literature indicate that the surface approach to learning taken by accounting students may cultivate their habits of academic dishonesty and encourage them to indulge in plagiarism.” (Xin, 2011, p. 19). If, as this literature seems to suggest, students adopting a surface approach to learning are more likely to adopt an approach to assessment that sees cheating as ethically unproblematic, the object of higher education becomes one of moving students from the surface and passive approaches to learning which characterize their entry behavior toward increasingly sophisticated levels of autonomy and deep levels of learning as they move toward the end of their undergraduate program.

However, while the concept of surface and deep learning has been widely adopted across higher education, it should be noted that the terms are not uncontested (Peters and Jones, 2007; Howie and Bagnall, 2015). Furthermore, Haggis (2003) paper calls the whole idea of the uncritical acceptance of the surface learning/deep learning dichotomy into question. While it may be possible to critique the terms deep and surface approaches to learning as meaning more to academics than to students, it does seem to be the case that there is a correlation between the approaches students take toward their studies, regardless of the labels we might wish to attach to them, and their predisposition toward cheating or not cheating in academic assessments. Thus, we formulate the following hypotheses:

H4: Surface Approach may facilitate Individual Cheating (H4a) and be positively associated with both Machiavellianism dimensions of Amoral Manipulation (H4b) and Desire for Status (H4c), thus facilitating the process through which Machiavellianism influences Individual Cheating. Conversely, Deep Approach may inhibit Individual Cheating (H4d) and be negatively associated to both Amoral Manipulation (H4e) and Desire for Status (H4f), thus inhibiting the process through which Machiavellianism influences Individual Cheating.

From these hypotheses, it is clear that students, whose approach to learning is dominated by the intention to get the task out of the way with minimum trouble and to achieve a minimal pass, may harbor a cynical view of education which may foster the recourse to cheating behavior as a shortcut to achieve their minimal academic goals. Conversely, students whose approach to learning arises from a felt need to engage the task appropriately and meaningfully, try to use the most appropriate cognitive activities for handling the academic task, and thus are less probable to indulge in academic cheating. Thus, a corollary of H4 is the following:

*H4g: Machiavellianism dimensions have an indirect effect on Individual Cheating through the two different approaches to study*.

### Role of Moral Disengagement

Moral disengagement is a social cognitive construct embedded within Bandura’s (1991, 2016) moral agency theory and it refers to those mechanisms allowing individuals to engage in rule-breaking behaviors (Gini et al., 2014). In particular, Bandura (1991, 2016) identified eight self-serving cognitive maneuvers, referring to four loci, that can be activated to silence one’s own moral control and engage in acts not in line with norms and rules (whether personal and/or socially shared). At the behavioral locus, *moral justification, euphemistic labeling*, and *advantageous comparison* may allow individuals to cognitively reframe the meaning and the seriousness of their misacts, making them socially and morally acceptable. At the agency locus, *diffusion* and *dislocation of responsibility* may allow individuals to reduce the “dose” of their own responsibility or to translate it to someone else, weakening or obscuring the link between their intentions and their behavior. At the outcome locus, *distortion* and *minimization of consequences* would allow individuals to reduce the perceived effect of their misconduct, exonerating them from the actual consequences. Finally, at the victim/target locus, *attribution of blame* and *dehumanization* may allow individuals to recognize the victims themselves as the actual cause of the suffered misbehavior.

In the last decade, a large body of research has confirmed the role of moral disengagement as a proximal antecedent of a wide variety of deviant conducts across different contexts (e.g., clinical: Hyde et al., 2010; developmental: Shulman et al., 2011; and civic: Caprara et al., 2009) and stages of life (e.g., Detert et al., 2008; Moore, 2008; Fida et al., 2015). As previously mentioned, with a specific reference to cheating behavior, it has been recently shown the relevance of moral disengagement in facilitating the likelihood of engaging in unethical conducts in the academic system (Fida et al., 2016). Consistently with these evidences, we hypothesize that:

*H5: Moral Disengagement is positively related to Individual Cheating*.

In addition, recent studies have underlined the contribution that moral disengagement has in mediating the relationship between personality dispositions, and aggressive and deviant behaviors (e.g., Caprara et al., 2013, 2014). In particular, it has been claimed that moral disengagement: (a) should be considered as “the most powerful predictor and the most proxy determinant” (Caprara et al., 2013, p. 301) of such types of misconducts; (b) is directly affected by personality traits; and (c) mediates all the relationships between personality dispositions and deviant behavioral outcomes (Caprara et al., 2013).

Finally, when considering the complex-posited network of relationships, it is also important to take into account those studies examining the association between motivational individual dimensions and moral disengagement. These contributions underlined that motivations associated to personal utility (e.g., financial gains) and to the affirmation of one’s own status (e.g., power values) foster the activation of moral disengagement, whereas those associated to personal development (e.g., self-realization) and to the preservation of everyone’s wellbeing (e.g., self-transcendence) contrast it (e.g., Paciello et al., 2013; Baron et al., 2015). Furthermore, some “immoral” goals may promote moral disengagement as a mean to rationalize and legitimize those unethical behavioral strategies serving the achievement of one’s own results (e.g., Beu and Buckley, 2004; Barsky, 2008). Hence, in line with this literature, we hypothesize that:

H6: Moral Disengagement is positively influenced by Amoral Manipulation (H6a) and Desire for Status (H6b). Moreover, Moral Disengagement also mediates the relationship between Machiavellianism dimensions and Individual Cheating (H6c).

H7: Moral Disengagement is positively influenced by Surface Approach (H7a), and negatively influenced by Deep Approach (H7b) to learning. Considering the hypothesized effects of Machiavellianism dimensions on Deep and Surface approaches, as a corollary of this hypothesis we expect that Machiavellianism dimensions have an indirect effect on Moral Disengagement through the two different approaches to study (H7c).

## Materials and Methods

### Participants and Procedure

A sample of 223 undergraduate psychology students took part in the study. Most participants were female (68.2%), with a mean age of 21.72 years (*SD* = 3.68). In terms of students’ family educational level, 53% of their mothers and their fathers completed senior high school, and 36% of their mothers and 31% of their fathers have a university degree.

Students were informed that participation was voluntary and that the research was not commissioned by the university they were enrolled in. In addition, a research team member clarified that students’ responses would be kept confidential and anonymous. Finally, students were informed that individuals’ data would not have been shared with anyone for any reason and that data would always have been reported in an aggregate form. They completed a paper and pencil questionnaire individually in a collective administration during a class after having signed the informed consent and received course credits for the participation in the research. The ethics committee of the university to which the first author is affiliated approved the study.

### Measures

#### Machiavellianism

The MPS (Dahling et al., 2009) was used. The MPS comprises four sub-scales: (1) *Amoral Manipulation* (five items, e.g., “I am willing to sabotage the efforts of other people if they threaten my own goals”); (2) *Desire for Control* (three items, e.g., “I like to give the orders in interpersonal situations”); (3) *Desire for Status* (three items, e.g., “I want to be rich and powerful someday”); and (4) *Distrust of Others* (five items, e.g., “I dislike committing to groups because I don’t trust others”). Preliminary EFA confirmed the four-factor structure of the questionnaire also in the Italian sample. Cronbach’s coefficient alpha of internal consistency for these four subscales was, respectively, 0.70, 0.76, 0.80, and 0.76. Items 2 and 5 of the Amoral Manipulation scale were no longer included as manifest indicators for further analyses, since they showed low factor loadings in the EFA target factor (i.e., <0.30): after this elimination factor loadings ranged from 0.31 to 0.97. Consistent with our premises, only Amoral Manipulation and Desire for Status dimensions of MPS will be further considered for the present study.

#### Approaches to Learning

The Revised Two Factor Study Process Questionnaire (R-SPQ-2F; Biggs et al., 2001) was used. It assesses two approaches to learning: *Deep Approach* and *Surface Approach*, each consisting of both motive and strategy. In particular, “surface motive” refers to the fear of failure and the desire to complete one’s own course of study (e.g., “My aim is to pass the course while doing as little work as possible”), and “surface strategy” underlies rote learning of facts and ideas, and focuses on task components in isolation (e.g., “I only study seriously what’s given out in class or in the course outlines”). In contrast, “deep motive” refers to the interest in subjects and personal understanding (e.g., “I feel that virtually any topic can be highly interesting once I get into it”) and “deep strategy” reflects the tendency to relate ideas to evidence and integrate material across courses (e.g., “I test myself on important topics until I understand them completely”). Italian form of the R-SPQ-2F includes 20 items with a five-point Likert scale ranging from 1 = “never or rarely true for me” to 5 = “always or almost always true for me.” Preliminary EFA confirmed the two-factor structure of the questionnaire also in the Italian sample. Factor loadings ranged from 0.39 to 0.71. Internal consistency in the current sample was 0.83 and 0.79 for Deep Approach and Surface Approach dimensions, respectively.

#### Academic Moral Disengagement

This construct was assessed by a 15-item Likert scale published by Farnese et al. (2011), generated on the basis of the general Moral Disengagement scale (Bandura et al., 1996) and a set of interviews to academic students. This scale assesses students’ proneness to moral disengagement of different forms of detrimental conduct in the academic context. Response options were presented in a five-point format, ranging from 1 = “not at all true” to 5 = “completely true.” Preliminary EFA on our data revealed a two-factor solution. In particular, the first factor (nine items) was mainly related to mechanisms of diffusion and displacement of responsibility, and advantageous comparison, identifying in common peers’ practices the origin of students’ misconducts (e.g., “Ask a colleague for a suggestion during an exam is just a request for solidarity”). The second factor (six items) was mainly related to mechanisms of attribution of blame and distortion of consequences, identifying in professors and university the origin of students’ misconducts (e.g., “If the dishonest students are not sanctioned by the university it is normal for a student to cheat”). Factors were correlated 0.53 (*p* < 0.001). Internal consistency reliability in the current sample was 0.84 for the first factor and 0.68 for the second.

#### Cheating Behavior

A 10-item Likert scale specifically developed by Farnese et al. (2011) was used. This scale assesses the frequency of engaging in academic deviant behaviors (e.g., “To copy sections of texts or articles taken from the Internet”) both for participants and their peers. Accordingly, each item describing a specific behavior was presented twice, asking students to indicate on a five-point response format, ranging from 1 = “never” to 5 = “always,” how frequently they and their colleagues have engaged in it. Preliminary EFA evidenced the presence of two different clusters of items, related, respectively, to conducts acted by the respondent (*Individual Cheating*) and conducts acted by peers (*Normative Cheating*). Factors were correlated 0.40 (*p* < 0.001). Internal consistency reliability in the current sample was 0.75 and 0.86 for Individual Cheating and Normative Cheating, respectively.

The two scales of Academic Moral Disengagement and of Cheating Behavior were developed in Italian (Farnese et al., 2011), hence no adaptation was required. The two scales of Machiavellianism and Approaches to Learning were translated into Italian from the English version using the standard translation-back-translation procedure recommended by Brislin (1980). The correspondence of the original and the back-translated items was then verified by the authors.

### Data Analysis

Since all constructs with the exception of Amoral Manipulation and Desire for Status were assessed by more than five items, we implemented a partially disaggregated approach in which latent factors were defined using parcels (i.e., the sum or the average of several items measuring the construct; Coffman and MacCallum, 2005). Specifically, since both Deep and Surface Approach sub-scales were monodimensional, for each of these two constructs three parcels were constructed using the *item-to-construct balance* strategy (Little et al., 2002) by examining the item-to-construct relationships, relying on factor loadings showed by EFA models at the item level. The same approach was used also for defining parcels of each set of items related to Individual Cheating Behavior and Normative Cheating. Since Academic Moral Disengagement items gave rise to two correlated factors, parcels for this construct were defined by factor scores derived from previous EFA conducted on the whole set of items. This approach is consistent with the so-called *homogenous parceling* strategy used for defining parcel scales when there is a global high-order construct encompassing different first-order factors which are defined directly by items (Coffman and MacCallum, 2005). Finally, since Amoral Manipulation and Desire for Status scales were assessed by a limited number of items, latent variables were defined using their corresponding items as manifest indicators. Thus, the final measurement model was a combination of total and partial disaggregation approaches to measurement model specification (Bagozzi and Heatherton, 1994).

From an analytical standpoint, we examined descriptive statistics and missing data of the manifest indicators (parcels or items) included in the measurement model part. Although we have no theoretical or methodological reasons to suspect that our data were missing not at random, we tested empirically this condition by means of the Little’s (1988), evaluating the null hypothesis that data were missing completely at random and they were unrelated to data values.

After this preliminary phase, the measurement model for the full set of data was examined using a Confirmatory Factor Analysis (CFA) positing seven correlated factors. Since the stem of the corresponding items used to measure both Individual Cheating and Normative Cheating were exactly the same and they were grouped with an identical parceling scheme, three pairs of correlations among their uniquenesses were specified *a priori* (Gerbing and Anderson, 1984). The posited CFA model was statistically compared with an alternative model where all manifest indicators loaded onto a single latent variable (i.e., Harman’s single-factor test) in order to evaluate the discriminant validity of the constructs under investigation. CFAs and further structural equation models were tested with *Mplus* v.8 (Muthén and Muthén, 1998-2017) and evaluated in terms of fit with the observed data using different indices: (i) χ^2^-test (if not statistically significant, the model fits perfectly the data); (ii) root mean square error of approximation (RMSEA; Steiger, 1990; if ≤0.05, the model shows a good fit); (iii) comparative fit index (CFI; Bentler, 1990; if ≥0.90, the model shows an acceptable fit); and (iv) standardized root mean squared residual (SRMR; Hu and Bentler, 1999; if ≤0.08, the model shows an acceptable fit).

Once the goodness of fit of the measurement model was established, the model depicted in **Figure 1** was tested using a two-step approach (see Klein and Moosbrugger, 2000). In a first step, the model was tested without including the latent interaction term (Model 1). Given the complexity of the structural model and consistent with Ullman (2006, see also Bentler, 2006), non-significant structural paths were evaluated with the multivariate Wald test, where the null hypothesis is that the set of parameters under scrutiny do not add useful information to the model, so they can be dropped from the final model. Once Model 1 was trimmed, the latent interaction term related to the hypothesized moderation of Normative Cheating on the Amoral Manipulation-Individual Cheating relationship (H3) was introduced (Model 2). Specifically, Model 2 was tested using full information maximum likelihood (FIML) with robust standard error (MLR) with numerical integration (Muthén and Muthén, 1998-2017). Besides scaling the latent variable by fixing the first factor loading to 1.0 as in Model 1, Model 2 was also retested by changing the scaling method (i.e., fixing latent variances to unity) and results were then compared in order to avoid potential biases in estimates dependent from the chosen latent scaling method (Gonzalez and Griffin, 2001). Since models positing latent interactions cannot be evaluated in terms of overall fit with common indices (e.g., RMSEA and CFI) because means, variances, and covariances do not represent sufficient statistics for model estimation, Model 2 was compared with Model 1 by means of several information criteria (see Kline, 2016), as the Akaike Information Criterion (AIC), the Bayesian Information Criterion (BIC), and the sample size-adjusted BIC (SABIC). Models showing lowest information criteria values should be preferred. Latent interaction was evaluated both statistically and graphically (Aiken and West, 1991).

As can be noted, the model depicted in **Figure 1** posits also different mediational paths. Since common procedures for interpreting the significance of indirect effects (e.g., bootstrapped confidence intervals, see MacKinnon et al., 2007) are not available when parameters are estimated along with numerical integration (see Muthén and Muthén, 1998-2017), as in our Model 2, we relied on distribution-of-product method and the associated 95% confidence intervals for their interpretation (Tofighi and MacKinnon, 2011).

## Results

### Preliminary Analyses

**Table 1** shows descriptive statistics for the observed variables (items and parcels) included in the CFA and full-SEM models that will be discussed in the present study. As can be noted, some of them exceed the value of |1| for univariate skewness and kurtosis, suggesting weak departure of some items and parcels from univariate normality. Thus, CFAs and structural equation models have been estimated using MLR (see Muthén and Muthén, 1998-2017), which corrects the χ^2^ for non-normality (Yuan and Bentler, 2000).

**Table 1:** Descriptive statistics for parcels and items.

	Mean	Standard deviation	Skewness	Kurtosis
**Individual Cheating**				
IND_CHEAT_P1	2.51	0.77	0.71	0.26
IND_CHEAT_P2	2.71	0.79	0.42	–0.02
IND_CHEAT_P3	1.65	0.55	1.75	2.85
**Normative Cheating**				
NORM_CHEAT_P1	3.68	0.86	0.20	–0.37
NORM_CHEAT_P2	3.69	0.78	0.26	–0.37
NORM_CHEAT_P3	3.28	0.90	0.17	–0.51
**Academic Moral Disengagement**				
AMD_FS_1	0	0.94	0.65	0.10
AMD_FS_2	0	0.88	0.38	–0.25
**Deep Approach**				
DEEP_APPR_P1	3.15	0.70	–0.18	–0.22
DEEP_APPR_P2	3.64	0.72	–0.54	0.53
DEEP_APPR_P3	2.99	0.71	0.17	–0.47
**Surface Approach**				
SURF_APPR_P1	2.28	0.81	0.46	–0.16
SURF_APPR_P2	1.66	0.60	0.93	0.32
SURF_APPR_P3	1.90	0.64	0.82	0.76
**MACH Desire for Status**				
DES_FOR_STAT_9	2.50	1.15	0.17	–1.13
DES_FOR_STAT_10	2.57	1.06	0.06	–0.99
DES_FOR_STAT_11	2.70	1.14	0.05	–0.88
**MACH Amoral Manipulation**				
AMOR_MANIP_1	2.12	0.99	0.67	–0.28
AMOR_MANIP_3	2.13	0.99	0.69	–0.13
AMOR_MANIP_4	1.53	0.72	1.06	–0.02

A preliminary check of missing data was performed on all the items and parcels considered in this study. While 99% of the sample has no missing data, three participants (1% of the total sample) have three missing data points (one participant) or a single missing value (two participants). Little’s MCAR test was not significant, $\chi_{\mathrm{(36)}}^{2}$ = 47.08 with *p* = 0.10, suggesting that the few missing data points were not related to any study indicator. Overall, missing data analysis provided support for the adoption of FIML (Arbuckle, 1996) to handle missing data in the following SEM analyses.

**Table 2** presents descriptive statistics and zero-order correlations for the study variables computed as scale means. Individual Cheating is moderately and positively correlated with Normative Cheating, Academic Moral Disengagement, and Amoral Manipulation, while it shows a weak positive correlation with Surface Approach and a weak negative correlation with Deep Approach. Normative Cheating exhibits weak positive correlations with Surface Approach, Academic Moral Disengagement, and Amoral Manipulation, which in turn is moderately and positively associated with Amoral Manipulation and Surface Approach, and shows a weak negative correlation with Deep Approach. Moreover, Academic Moral Disengagement shows a positive (albeit weak) correlation with Desire for Status. Surface and Deep Approaches are moderately and negatively associated, and while Deep Approach shows a weak negative correlation with Amoral Manipulation, this latter variable is moderately and positively associated with Surface Approach. Finally, Desire for Status is weakly and positively associated with Amoral Manipulation.

**Table 2:** Descriptive statistics and zero-order correlations among the study variables.

	*M*	*SD*	*K*	*SK*	1	2	3	4	5	6	7
1. Individual Cheating	1.69	0.43	1.00	1.30	1						
2. Normative Cheating	2.81	0.61	0.25	–0.59	0.43**	1					
3. Academic Moral Disengagement	2.02	0.52	0.44	–0.13	0.47**	0.17*	1				
4. Deep Approach	3.23	0.61	0.01	–0.26	–0.32**	–0.07	–0.24**	1			
5. Surface Approach	1.94	0.56	0.58	0.10	0.35**	0.20**	0.43**	–0.39**	1		
6. Desire for Status	2.59	0.94	0.12	–0.83	0.03	–0.08	0.14*	0.13	0.04	1	
7. Amoral Manipulation	1.83	0.64	0.57	–0.23	0.40**	0.13*	0.49**	–0.17**	0.37**	0.29**	1

*M, mean; *SD*, standard deviation; *K*, kurtosis; *SK*, skewness. The mean of Academic Moral Disengagement parcels is 0 because they are factor scores. ^∗∗^*p* < 0.01, ^∗^*p* < 0.05.*

### Measurement Model

The hypothesized CFA model showed an excellent fit to the data: $\chi_{\mathrm{(df = 146,N = 223)}}^{2}$ = 169.57, *p* = 0.088, RMSEA = 0.027 (90% CI = 0.000–0.043, *p* = 1.00), CFI = 0.987, TLI = 0.983, SRMR = 0.046. The seven factors were significantly loaded by the intended manifest indicators, providing support to the theoretical constructs. Factor loadings of the tested measurement model are presented in **Table 3**, along with those of Model 1 and Model 2 (see below). The correlations among the factors ranged from -0.49 (Surface Approach with Deep Approach) to 0.69 (Amoral Machiavellianism with Academic Moral Disengagement). Only two out of the three pairs of correlated residuals among Individual and Normative Cheating parcels were significant. However, also the non-significant covariance among residuals was maintained in order to properly control for their shared element not attributable to substantive variance (see Cole et al., 2007).

**Table 3:** Factor loadings from the measurement model, Model 1 and Model 2.

	Measurement model	Model 1	Model 2
**Individual Cheating**			
IND_CHEAT_P1	0.819	0.811	0.823
IND_CHEAT_P2	0.806	0.794	0.763
IND_CHEAT_P3	0.657	0.644	0.658
**Normative Cheating**			
NORM_CHEAT_P1	0.913	0.911	0.914
NORM_CHEAT_P2	0.825	0.824	0.821
NORM_CHEAT_P3	0.858	0.862	0.860
**Academic Moral Disengagement**			
AMD_FS_1	0.904	0.905	0.909
AMD_FS_2	0.800	0.800	0.796
**Deep Approach**			
DEEP_APPR_P1	0.857	0.854	0.859
DEEP_APPR_P2	0.736	0.735	0.732
DEEP_APPR_P3	0.738	0.739	0.737
**Surface Approach**			
SURF_APPR_P1	0.814	0.814	0.812
SURF_APPR_P2	0.705	0.703	0.704
SURF_APPR_P3	0.620	0.620	0.621
**MACH Desire for Status**			
DES_FOR_STAT_9	0.567	0.568	0.569
DES_FOR_STAT_10	0.880	0.875	0.876
DES_FOR_STAT_11	0.837	0.840	0.839
**MACH Amoral Manipulation**			
AMOR_MANIP_1	0.689	0.691	0.702
AMOR_MANIP_3	0.918	0.917	0.888
AMOR_MANIP_4	0.381	0.381	0.386

*Factor loadings are presented in a completely standardized metric.*

Finally, the single-factor model yielded a very poor fit: $\chi_{\mathrm{(36)}}^{(df = 167,N = 223)}$ = 1,203.64, *p* < 0.001, RMSEA = 0.167 (90% CI = 0.158-0.166, *p* < 0.001), CFI = 0.422, TLI = 0.342, SRMR = 0.139. Compared with the hypothesized measurement model, the single-factor model fit worst the observed data, with a scaled Δ$\chi_{\mathrm{(\Delta df=21)}}^{2}$ = 998.96, *p* < 0.001. This result supports the discriminant validity of the study measures and constructs.

### Trimming the Structural Model

Once established the goodness of fit of the overall measurement model, we tested Model 1 (i.e., the hypothesized model in **Figure 1** not including the latent interaction). This model showed an adequate fit to the data: $\chi_{\mathrm{(df = 151,N = 223)}}^{2}$ = 179.80, *p* = 0.055, RMSEA = 0.029 (90% CI = 0.000-0.044, *p* < 0.001), CFI = 0.984, TLI = 0.980, SRMR = 0.064. However, some structural effects (specifically, those related to H1b, H4a, H4c, H6b, and H7b paths reported in **Figure 1**) were not statistically significant. Accordingly, we tested with the multivariate Wald test whether these parameters could be dropped from the model without losing information. This resulted not significant: $\chi_{\mathrm{(df=5)}}^{2}$ = 3.76, *p* = 0.585, suggesting that these parameters can be reasonably eliminated. As a further check, we statistically compared Model 1 with its revised version, where the non-significant paths were fixed at 0. This comparison was not significant, with a Δ$\chi_{\mathrm{(\Delta df=5)}}^{2}$ = 4.55, *p* = 0.473, additionally confirming that dropping non-significant paths do not result in a worst model. Thus, the revised version of Model 1 was elected as the final.

**FIGURE 2:**
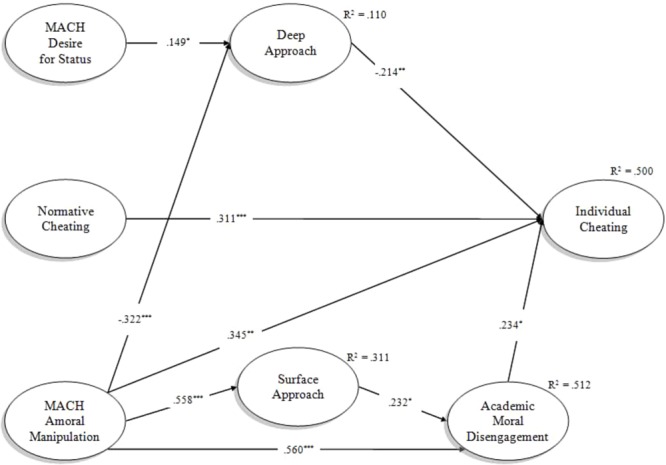
Standardized estimates from the “revised” Model 1. For sake of clarity, correlations were not reported. ^∗∗∗^*p* < 0.001, ^∗∗^*p* < 0.01, ^∗^*p* < 0.05.

The *trimmed* Model 1 fitted the data well: $\chi_{\mathrm{(df = 156,N = 223)}}^{2}$ = 184.38, *p* = 0.060, RMSEA = 0.029 (90% CI = 0.000-0.044, *p* < 0.001), CFI = 0.984, TLI = 0.981, SRMR = 0.064, and the related standardized estimates are reported in **Figure 2**. Although correlations are not reported in **Figure 2**, Amoral Manipulation and Desire for Status showed a weak positive latent correlation (0.164, *p* < 0.05) and Deep Approach and Surface Approach showed a moderate negative latent correlation (-0.407, *p* < 0.001). In line with the corresponding hypotheses, the effect of Amoral Manipulation on Individual Cheating (H1a) was positive and significant (0.345, *p* < 0.01), so was the direct path of Normative to Individual Cheating (H2, 0.311, *p* < 0.001). Results supported also H4b, since the effect of Amoral Manipulation on Surface Approach was significant and in the expected direction (0.558, *p* < 0.001). Moreover, Deep Approach showed a significant, negative impact on Individual Cheating (-0.214, *p* < 0.05), confirming H4d. With regards to H4e, Amoral Manipulation was significantly and negatively associated to Deep Approach (-0.322, *p* < 0.001) as anticipated. Contrary to our expectations, the direct effect of Desire for Status on Deep Approach (H4f) showed a positive sign (0.149, *p* < 0.05): the interpretation of this result will be addressed in the section “Discussion.” As expected, Academic Moral Disengagement significantly influenced Individual Cheating (H5), with a standardized direct effect of 0.234 (*p* < 0.05). Amoral Manipulation impacted significantly Moral Disengagement (H6a) in the expected direction (0.560, *p* < 0.001). Finally, Surface Approach contributed significantly in explaining Academic Moral Disengagement scores (0.232, *p* < 0.05), as posited in H7a. Overall, the following percentages of latent variance were explained by proximal and distal determinants posited by the model: 11.0% of Deep Approach, 31.1% of Surface Approach, 51.2% of Academic Moral Disengagement, and 50.0% of Individual Cheating.

### Interactive and Mediation Effects

**FIGURE 3:**
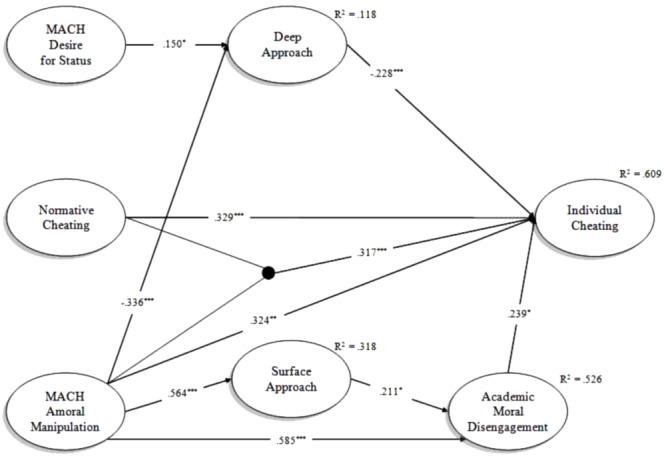
Standardized estimates from Model 2. For sake of clarity, correlations were not reported. ^∗∗∗^*p* < 0.001, ^∗∗^*p* < 0.01, ^∗^*p* < 0.05.

Once the trimmed Model 1 was established, the latent interaction posited by H3 was introduced and tested in Model 2. The information criteria values were AIC = 9,026.19, BIC = 9,278.32, and SABIC = 9,043.81 for Model 1 and AIC = 9,006.79, BIC = 9,262.32, and SABIC = 9,024.64 for Model 2. Since all information criteria values for Model 2 were lower, it should be preferable to Model 1. Thus, the inclusion of the latent interaction did not result in a worse overall model fit. As a further check, latent variable scaling was also defined by fixing latent variances to unity and freeing the first loading within each factor, in order to detect potential differences in results attributable to different scaling methods (Gonzalez and Griffin, 2001) and, consequently, in the interpretation of the latent interaction (see Maslowsky et al., 2015). In this case, no differences in estimates were detected between the default method of latent variable scaling (i.e., free latent variances and first loading fixed to unity) and the one described above. **Figure 3** presents standardized estimates from Model 2. As can be noted, coefficients of structural path closely resemble to those observed for Model 1 (**Figure 2**). Also the latent correlations between Amoral Manipulation and Desire for Status (0.167, *p* < 0.05), and between Deep Approach and Surface Approach (-0.397, *p* < 0.001) were similar to those observed in the previous model.

**FIGURE 4:**
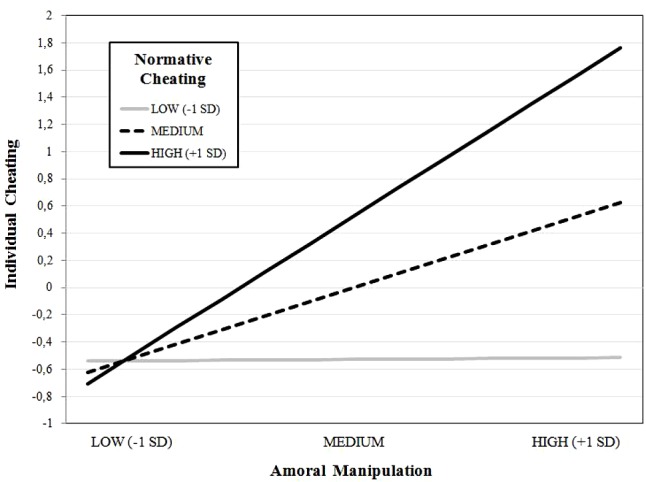
Plot of the latent interaction between Amoral Manipulation and Normative Cheating on Individual Cheating.

The latent interaction of Normative Cheating on the direct effect of Amoral Manipulation over Individual Cheating (H3) was significant (0.317, *p* < 0.001). Noteworthy, the inclusion of the latent interaction increased the overall proportion of explained variance in Individual Cheating of about 11.0%, compared with the model where the latent interaction term was not included. The plot of the latent interaction is reported in **Figure 4**. It shows that when Normative Cheating is low, the effect of Amoral Manipulation on Individual Cheating is absent; on the contrary, when Normative Cheating is medium the former impact on the latter and this effect is even stronger when Normative Cheating is high.

Finally, specific and total indirect effects were examined. Overall, the total indirect effect of Amoral Manipulation on Individual Cheating was significant (0.231, *SE* = 0.089, 95% CI 0.023–0.280). Moreover, also two specific indirect effects of Amoral Manipulation on Individual Cheating were significant. In particular, Amoral Manipulation showed a significant indirect impact on Individual Cheating via Deep Approach (H4g, 0.076, *SE* = 0.026, 95% CI 0.031–0.131), and via Academic Moral Disengagement (H6c, 0.138, *SE* = 0.066, 95% CI 0.012–0.271). Finally, the specific indirect effect of Amoral Manipulation on Academic Moral Disengagement via Surface Approach was significant (H7c, 0.119, *SE* = 0.055, 95% CI 0.015–0.231). None of the other mediation effects resulted statistically significant.

## Discussion, Limits, and Conclusion

This paper examined a complex model aimed at understanding cheating behaviors in the academic context. In particular, different constructs were considered, integrating different theoretical frameworks related to the study of academic cheating, within a common overarching nomological network. As hypothesized, academic cheating is primarily sustained by the personality dimension of Machiavellianism, and more specifically, by its Amoral Manipulation facet (as per H1a). Previous studies resulted in contradictory findings regarding the relationship between Machiavellianism and academic cheating, and this may be due to the way in which Machiavellianism has been operationalized. Investigating its impact disregarding its distinct features it is likely to provide a blurred picture. Indeed, Machiavellianism is a complex dimension, comprising different facets or “sub-dimensions” not equally relevant for understanding the engagement in academic cheating. In our study, we focused the attention on two of the four dimensions theorized by Dahling et al. (2009): Desire for Status and Amoral Manipulation. The former resulted to be not significant, implying that the component of Machiavellianism related to the desire for achieving external goals, such as status and success, is not relevant for the engagement in academic cheating, neither directly nor through the mediation of other variables, thus disconfirming our hypothesis H1b.

On the contrary, Amoral Manipulation resulted to be a key dimension, implying that the component of Machiavellianism related to the tendency to manipulating others and to the lack or disregard of conventional morality, with the aim of reaching one’s own goals, is particularly important to understand the engagement in cheating behavior. Amoral Manipulation influenced individual academic cheating not only directly, but also through the mediation of Surface Approach to study and of Moral Disengagement. Although these two latter variables refer to theoretical models different from the personality traits approach, they contributed to explain the engagement in academic cheating in combination with Machiavellianism. In this regard, a net of relationships conducive to academic cheating is made possible when Amoral Manipulation reinforces the easy access to mechanisms of moral disengagement in order to silence one’s own moral control when breaking norms and rules intended to regulate academic endeavors. This process may be even exacerbated when Amoral Manipulation facilitates student’s surface approach to learning (as demonstrated by our hypothesis H4b), characterized by the intention to get the task out of the way with minimum trouble and to achieve a minimal pass, which in its turn reinforces the adoption of moral disengagement mechanisms (as per H7a). The resort to academic cheating behaviors is further supported by the role that Amoral Manipulation has in hindering students’ deep approach to learning, characterized on the contrary by a genuine need to engage appropriately and meaningfully in academic tasks. Moreover, Amoral Manipulation effect on academic cheating is exacerbated when students are in an academic context where they perceive academic cheating being considered as “a norm” by their peers, as demonstrated by our interaction hypothesis H3.

It is noteworthy that the relationship with academic cheating is not shared by the other component of Machiavellianism considered in this study: the Desire for Status. In particular, contrary to our expectations, this dimension did not result significantly associated with academic cheating neither directly nor through any other variable included in the model. In addition, it showed an unexpected positive association with deep approach to learning. As mentioned above, deep approach is concerned with a genuine interest in academic subjects and personal understanding, coupled with appropriate cognitive activities for handling academic task. In our study, this approach appears to be reinforced by the Desire for Status feature of Machiavellianism. In the specific context where this study has been conducted, those students who strive for success are motivated to adopt a deep approach to study, which may be conducive not only to excellent grades but especially to a solid background in the academic subjects, which represents the building block of success in students’ future professional career. In fact, the Desire for Status feature of Machiavellianism did not result associated neither with academic cheating nor with surface approach, contrarily to what has been hypothesized in our nomological network. These results are coherent with the idea that, at least in our study, desire for status is a positive feature more concerned with strategically “climbing the social ladder” rather than with manipulative tactics aimed at looking for a way out in the academic context. Overall, the model showed that different individual characteristics contribute, in a complex interrelation, to students’ cheating behavior. Specifically, Amoral Manipulation personality trait, on the one side enhances surface approach to learning and enacts moral disengaged mechanisms; on the other side, it decreases Deep Approach to learning. Moreover, it further interacts with perception of peers’ academic misconducts. All these paths pave the way to the adoption of cheating behavior.

In our model not only Machiavellianism but also the other constructs we examined resulted relevant with regard to the explanation of academic cheating above and beyond what explained by Machiavellianism. Normative cheating, measured as participants’ perception of the frequency with which their colleagues engage in academic cheating behaviors, represents a cultural milieu in which the adoption of academic cheating can be considered as an implicit norm, or at least as a tolerated and plausible behavior. As evidenced in our model, the effect of this variable is substantially independent from the effect of all the other variables.

The other construct contributing to academic cheating is the deep approach to learning (as per H4d). As we noted above, this construct has an inhibitory influence on academic cheating, above and beyond the reinforcing role of normative cheating, moral disengagement, and amoral manipulation. Students who prevalently adopt this approach are genuinely involved into the academic subjects, and see studying as a mean of personal fulfillment (Asikainen and Gijbels, 2017). These students may consider academic activities as essential to their personal growth and to their future professional identity, and cheating as dangerous for them. The lack of relationship between surface approach and individual cheating was surprising and contrary to what hypothesized in our model. Indeed, the significant positive zero-order correlation between them disappears when controlling for moral disengagement and amoral manipulation. Thus, while the adoption of a deep approach contributes to prevent from academic cheating, the adoption of a surface approach is not predictive of the adoption of cheating academic behavior.

Finally, academic cheating is legitimated by the resort to moral disengagement mechanisms. Noteworthy, this does not depend on the environment shared by the students. Hence, independently from what students’ perceive to be their colleagues behavior, they rely on moral disengagement when they engage in cheating behavior to deal with internal norms and standards which may be violated by the adoption of academic dishonest conducts.

From a practical perspective, the results of the net of relations examined in this research suggested that there are different pathways through which academic cheating can be addressed in order to try to reduce it. Considering that Amoral Machiavellianism resulted as the main source of academic cheating, one may question whether students must be selected on the basis of this trait: however, this can be considered both impractical (due to social desirability responding) and especially unethical. A much more reasonable approach based on the hypotheses demonstrated in our study is to capitalize on utilitaristic/opportunistic nature of Amoral Machiavellianism, on the role that normative cheating resulted to have in moderating the relation between Machiavellianism and academic cheating, as well as on the mediational role of moral disengagement and learning strategies.

Amoral Machiavellians will engage in dishonest and deviant behavior when this is expected to produce a benefit for them, while being associated with a marginal risk of being caught and sanctioned. A “reversed cost–benefit” ratio should be then promoted, in which the costs associated to cheating are making high and inevitable, and the potential benefits are reduced to a minimum. This may for instance imply enforcing a consistent code of Ethic in which the consequences of academic cheating for students who resort to it should be clearly prescribed. The system of monitoring and sanctions should be equally made known to all the students and should be applied consistently, with no exceptions. Besides directly contrasting the expected impact of amoral Machiavellianism on individual academic dishonesty, this might also discourage normative cheating, which as shown in the present study has a key moderating effect. As evidenced in **Figure 4**, in our model the effects of Machiavellianism on cheating are particularly exacerbated in contexts where cheating is highly normative, whereas when the adoption of cheating behaviors is not the norm, this relation disappears. On the basis of this result, academic institutions should encourage an environment where cheating is not the implicit norm (McCabe and Treviño, 1993). Academic honest behavior may be further reinforced by means of a meritocratic system (e.g., ethics awards) and of specific courses (or part of courses) devoted to ethical and deontological subjects (McCabe et al., 1999), aimed to promote the salience of moral standards and rules and, in turn, potentially contrast the activation of moral disengagement mechanisms. Indeed, discouraging the resort to moral disengagement mechanisms for justifying one’s own academic cheating may be conducive to a reduction in academic cheating. While disengagement mechanisms alter the meaning, the consequences, the seriousness of the unethical behavior, these forms of cognitive distortion can be challenged by reframing and restructuring the “false” beliefs underlying them (e.g., by underlying that the first victim of the cheating is the same cheater). Specific courses on the ethical and deontological aspects of the subjects and of the future professions may contribute to reduce the distorting aspect of the unethical beliefs (Hren et al., 2006). The concurring of these different measures may render clear to students high in Amoral Manipulation that *cheating does not pay*.

Moreover, these measures could have an effect in reducing cheating independently from Machiavellianism: according to our model, normative cheating is an important antecedent of individual cheating *per se* (in this regard see also McCabe and Treviño, 1997). Since we demonstrated the role of deep learning in contrasting academic cheating, this opens another important pathway on which academic institutions may capitalize. Indeed, academic institution may promote intervention in order to move students from surface and passive approaches to increasingly sophisticated levels of autonomy and deep approach to learning. For instance, this can be obtained by encouraging the adoption of active and interactive teaching methods and techniques such as case studies, group-based learning (e.g., use of group problem-solving exercises, group presentations, group assignments), cooperative learning, flipped classrooms, among others. More in general the adoption by teacher of a student-focused approach, aimed at bringing about conceptual change and intellectual development in students, has showed to be successfully promoting a deep learning approach in students.

This study presented some limitations. First, data are based on self-reports. Although our data on many variables were not found dramatically skewed in a negative sense, students may have consciously (or not) underestimated their actual perceptions and behaviors (Paulhus and John, 1998), especially in relation to “sensitive” aspects as Individual Cheating and Academic Moral Disengagement. Further investigations should collect these data also from other sources (e.g., institutional reports related to honor code violations) and informants (e.g., course mates and teachers). Second, results are based on cross-sectional data. Although we built a theoretically driven nomological net among our study variables, it is not possible to draw substantive conclusions in terms of causality. Future studies may capitalize on our results for conducting cohort studies in order to take a closer look to the dynamic processes leading students to cheat in academic contexts. Third, our sample was not representative of the actual university study population. Thus, findings of the present studies should be replicated on other groups of students, possibly related to different cultural contexts.

## Ethics Statement

This study was carried out in accordance with the recommendations of the Ethic Committee of the Department of Psychology, Sapienza University of Rome. The protocol was approved by the Department of Psychology Ethics Committee, Sapienza University of Rome. All subjects gave written informed consent in accordance with the Declaration of Helsinki.

## Author Contributions

CB contributed on all sections of the paper. In particular he took care of the theoretical framework, to the literature review related to Machiavellianism, to the methods, to discussion and conclusion, and to the definition of the data analytical strategy. MF contributed on the literature review related to context as a source of norms for cheating, and added her contribution to the writing of the sections “Materials and Methods”, “Results,” and “Discussion.” CT contributed on the literature review related to academic cheating, and added his contribution to the writing of the sections “Materials and Methods”, “Results,” and “Discussion.” RF and MP contributed on the literature review related to Moral Disengagement, and added their contribution to the writing of the sections “Materials and Methods”, “Results,” and “Discussion.” VG contributed mainly to the data analysis, and to the writing of the section “Results,” and added his contribution to the writing of the section “Discussion.” PL contributed on the literature review related to Surface and Deep Approach to Learning, and added his contribution to the writing of the section “Discussion.”

## Conflict of Interest Statement

The authors declare that the research was conducted in the absence of any commercial or financial relationships that could be construed as a potential conflict of interest.
